# Screening and Optimizing Antimicrobial Peptides by Using SPOT-Synthesis

**DOI:** 10.3389/fchem.2017.00025

**Published:** 2017-04-12

**Authors:** Paula M. López-Pérez, Elizabeth Grimsey, Luc Bourne, Ralf Mikut, Kai Hilpert

**Affiliations:** ^1^TiKa Diagnostics LtdLondon, UK; ^2^Institute for Infection and Immunity, St. George's University of LondonLondon, UK; ^3^Karlsruhe Institute of Technology (KIT), Institute for Applied Computer Science (IAI)Eggenstein-Leopoldshafen, Germany

**Keywords:** SPOT-synthesis, antimicrobial peptides, peptide synthesis, antimicrobial screening, peptide libraries, substitution analysis, multi-drug resistance, tethered peptides

## Abstract

Peptide arrays on cellulose are a powerful tool to investigate peptide interactions with a number of different molecules, for examples antibodies, receptors or enzymes. Such peptide arrays can also be used to study interactions with whole cells. In this review, we focus on the interaction of small antimicrobial peptides with bacteria. Antimicrobial peptides (AMPs) can kill multidrug-resistant (MDR) human pathogenic bacteria and therefore could be next generation antibiotics targeting MDR bacteria. We describe the screen and the result of different optimization strategies of peptides cleaved from the membrane. In addition, screening of antibacterial activity of peptides that are tethered to the surface is discussed. Surface-active peptides can be used to protect surfaces from bacterial infections, for example implants.

## Antimicrobials and microbial resistance

Since their introduction in the 1930's, antibiotics have been heralded as the wonder discovery of the twentieth century; in the 75 years since their initial introduction (Davies and Davies, 2010), the foundations surrounding the way physicians and health care practitioners care for patients has shifted from a focus on diagnostics, toward a more treatment focused approach that has saved millions of lives worldwide (Spellberg et al., 2011). Undoubtedly, access to efficient antibiotics is of critical importance to society, with numerous procedures including; organ transplants, orthopedic surgery and chemotherapy carrying high, if not prohibitive risk without the accessibility of these antimicrobial agents (Höjgård, 2012). In fact, in the aftermath of this indisputable success, in late 1960's US Surgeon General William H. Stewart stated: “it is time to close the book on infectious diseases and declare the war against pestilence won” (Spellberg et al., 2008). Unfortunately, these seven decades of medical advances and the effectiveness of any novel antibiotic is compromised by the relentless appearance of drug resistant organisms, exhibiting resistance against one or more antibiotic types (Spellberg et al., 2011). The (O'Neill, 2016) commissioned by the former UK Prime Minister David Cameron, estimates that as of 2014, 700,000 people die annually from anti-microbial resistance (AMR) bacterial infections, costing the US health care system alone, $20 billion (O'Neill, 2016). Even more worryingly, these numbers are set to rise, with an estimated 10 million people predicted to succumb to AMRs by 2050 at an increasing global cost of $100 trillion. While the trends of AMR are difficult to predict, it is estimated that the death toll could be as high as one person every 3 s if this issue is not immediately addressed (O'Neill, 2016). While these statistics are frightening, this fate could be avoided by implementing certain interventions including: global public awareness campaigns, a reduction in the number of unnecessary prescriptions, dramatic reduction and restrictions of antibiotics in agriculture, development of rapid diagnostics and most importantly, the development of novel antimicrobial agents (Spellberg et al., 2011; O'Neill, 2016).

A large proportion of pharmaceutical companies have lost interest in the antibiotic market despite an overall increase in their research and development (R&D) budgets (A.A.D., 2004). In fact, as of 2013, only four big pharmaceutical companies have antibiotic programmes remaining (Fair and Tor, 2014). While the reasons for this are multifactorial, one main reason is that is has become substantially more difficult than it once was to develop a new antimicrobial, particularly against AMR Gram-negative bacteria. In addition, priced at a maximum of $1,000–$3,000 per course, antibiotics hold a very small profit margin in comparison to pharmaceuticals commonly used for long-term illness, which can reach in excess of $80,000 (Bartlett et al., 2013). Therefore, it is critical that pharmaceutical companies are provided an incentive to revitalize interest in antibiotic development.

## Antimicrobial peptides

Antimicrobial peptides (AMPs) are a diverse family of short length molecules, between 5 and 50 amino acids in length and with most possessing an overall net positive charge to their structure (Hancock and Patrzykat, 2002). These peptides have been gaining attention over recent years as one of the more promising alternatives to traditional antimicrobial drugs–they can display broad spectrum killing properties to Gram-positive and Gram-negative bacteria, fungi, viruses, parasites and even cancerous cells, are fast acting and have a decreased likelihood to induce pathogenic resistance as compared to traditional antimicrobial drugs (Marr et al., 2006).

Over 2,000 AMPs, both natural and lab-synthesized, have been documented and submitted to the Antimicrobial Peptide Database (APD3) (Wang et al., 2009; Zhao et al., 2013). In 2016, the APD3 contained 2,169 antibacterial and 959 antifungal peptides. AMPs can be found naturally in a huge variety of organisms—in animals, plants and even in fungi and bacteria (Leippe, 1999; Radek and Gallo, 2007). It is now understood that these molecules are a major part of the innate immune system of animals, with a large quantity of them localized to the organs and tissues most at risk of coming into contact with pathogens, such as the skin, airways, digestive tract and other epithelial surfaces and mucous membranes (Wiesner and Vilcinskas, 2010). Through release by immune cells, such as phagocytes and T-cells, AMPs are able to rapidly target and kill pathogenic threats (Diamond et al., 2009). They are playing a role in host inflammatory response by their action in modulating cytokine response subsequent to infection, as was recently shown for example when the AMP LL-37 decreased the cytokine response of human neutrophils after infection with *S. aureus* and *P. aeruginosa* (Alalwani et al., 2010). Conversely, AMPs have also shown the ability to upregulate immune response, such as human ɑ-defensins, produced in neutrophil cells, which have been shown to promote the production of pro-inflammatory cytokines (van Wetering et al., 1999).

The cationic nature of most AMPs means that they interact strongly with negatively charged molecules. Therefore, the selectivity of most AMPs relies on a fundamental difference in the architecture of mammalian and microbial membranes (Yeaman and Yount, 2003), allowing the peptide to selectively kill microorganisms (Matsuzaki, 2009). These include; differences in membrane composition, transmembrane potential and membrane polarization as well as other features including lipopolysaccharide (LPS), sterols, glycerides and peptidoglycan (Yeaman and Yount, 2003). The outside of bacterial membranes is rich in negatively charged phospholipids, such as: phosphatidylglycerol, phosphatidylethanolamine and cardiolipin (Zasloff, 2002). Further anionic molecules also exist in the bacterial cell wall, including lipoteichoic acids in the peptidoglycan of Gram-positive bacteria and LPS present in the outer membrane of Gram-negative bacteria (Matsuzaki, 2009; Guilhelmelli et al., 2013). In comparison, the negatively charged phospholipids of mammalian membranes usually reside in the inner leaflet of the bilayer (Zasloff, 2002), while the outermost leaflet, the layer exposed to the cytoplasm, is populated by the neutral phospholipids sphingomyelin, phosphatidylcholine, as well as sterols, such as cholesterol (Matsuzaki, 2009; Guilhelmelli et al., 2013). This difference in the charge of mammalian and microbial membranes means that AMPs show different affinities toward each respective membrane.

Another key variance between mammalian and microbial cells is the difference in the charge separation between the intra- and extracellular components of the cytoplasmic membrane. This transmembrane potential (Yeaman and Yount, 2003), dictated by the interactions between the negatively charged peptide and the phospholipid headgroups (Teixeira et al., 2012), is a result of the different rates and extents of proton flux over the cell membrane. In bacterial cells, this transmembrane potential is more negative, ranging from −130 mv to −150 mV in comparison to mammalian cells, with a range of −90 mv to −110 mV (Yeaman and Yount, 2003). Because cells with a more negative transmembrane potential facilitate membrane permeabilisation by encouraging cationic peptides to insert themselves into the membrane (Matsuzaki, 2009), cationic peptides can more easily permeabilise the bacterial cells, further providing AMPs with a means of selectivity toward microbial cells (Yeaman and Yount, 2003). A study performed by Matsuzaki supported this theory by demonstrating that the binding constant of the peptide tachyplesin is increased 200-fold when the membrane potential is as low as −120 mV (Matsuzaki et al., 1997; Yeaman and Yount, 2003).

Anionic AMPs can interact by amidation of the C-terminus (maxim H5) (Dennison et al., 2015), or through the formation of salt bridges by metal ions (Harris et al., 2009). Once this association has been completed, the AMP can be inserted into the membrane via the hydrophobic regions of its structure (Madani et al., 2011). AMPs can exert their antimicrobial effect by acting on the outside of their target at the cell membrane, or they can function by entering the cell and interacting with intracellular proteins or disrupting key processes, such as RNA- and DNA- synthesis.

For some AMPs a combination of extra- and intracellular activity is required for the death of their target (Koo et al., 2001). A major mechanism by which certain AMPs carry out their function on an intracellular level is by the inhibition of DNA, RNA and protein synthesis (Hilpert et al., 2010). Buforin 2, an AMP found in the stomach of Bufo gargarizans, the Asian toad, is able to penetrate the cell membrane via the action of a proline hinge and bind to DNA and RNA (Kobayashi et al., 2000; Xie et al., 2011), likely due to the similarity in sequence between buforin 2 and histone H2A's N-terminus (Cho et al., 2009). Indolicidin, isolated from bovine neutrophil granules, has been shown to bind to DNA where it blocks DNA-dependent enzymes, such as integrase from binding (Marchand et al., 2006). The central PWWP amino acid motif, a feature common to proteins involved in DNA binding (Hale and Hancock, 2007) has also shown importance in stabilizing the DNA structure once the peptide has attached (Ghosh et al., 2014). Attacin is able to contribute to destabilization of the membrane of *E. coli* by preventing the transcription of the omp gene, thereby blocking the synthesis of important outer membrane proteins (Carlsson et al., 1991). It was also described that proline-rich peptides can bind the chaperon DnaK and the ribosome (Krizsan et al., 2015; Knappe et al., 2016a). Short AMPs can interact with ATP and inhibit ATP depended enzymes critical for the survival of the bacteria (Hilpert et al., 2010). AMPs are also capable of preventing cell wall synthesis. Peptidoglycan is a major structural component of bacteria, being the main constituent of the cell wall in Gram-positive organisms and linked with the outer cell membrane in Gram-negative bacteria, providing protection and support. An important precursor of its synthesis is lipid 2, which has been shown to be the target of several AMPs including HND-1 (de Leeuw et al., 2010) and Cg-Defh-1 (Schmitt et al., 2010).

## Peptide libraries

Screening of compounds libraries is well established as a high-throughput method for detecting and studying interactions in both biological and chemical systems. Libraries can be composed of various types of molecules, ranging from small organic compounds to peptides, proteins and RNA/DNA. Many important processes in life are regulated by peptides of different size and complexity. Well-known examples are peptide hormones, like insulin, peptide neurotransmitters (e.g., opioid peptides) that influence pain and mood and peptides that influence digestion and vascular functions. There are peptides that support the immune system like (AMPs). In many biological toxins, from plants (fungi, phalloidin) to animals (honey bee, apamin), peptides play an important role (Jakubke and Sewald, 2009). Proteins play key roles in many cellular processes and in some cases can be recapitulated by shorter peptides taken from the primary protein sequences (although often with partial loss of activity). Peptides have come into the focus of the pharmacological industry not only based on high potency, but also their high selectivity and safety. The global peptide drug market was US$14.1 billion in 2011 and is estimated to reach US$25.4 billion in 2018 (Fosgerau and Hoffmann, 2015). Hence, peptide libraries can serve as valuable tools for identifying and optimizing biologically active compounds. Biological synthesized peptide libraries, such as phage, yeast, bacterial or ribosomal mRNA displays (Ullman et al., 2011) rely on (i) creating a diverse genetic library in which the phenotype (binding to target) of each member of the library is linked to its genotype (the encoding DNA or RNA), and (ii) an iterative cycle in which library members are selected for binding to a target, and then amplified (by replication in a host cell, or by copying of the encoded nucleic acid *in vitro*). These techniques allow the selection of highly active peptides due to several rounds of enrichment and a huge number of different peptide sequences (over 10^9^) that can be displayed.

However, even being nowadays possible (Tian et al., 2004), the use of non-natural amino acids is still difficult. Through optimization of the chemistry, automation and miniaturization of solid-phase peptide synthesis, chemical peptide libraries can be built using different solid supports (resin beads, pins, glass chips, tea bags, and cellulose membranes) not being restrictive to the use of gene-coded amino acids (Houghten, 1985; Weinberger et al., 1997; Tribbick, 2002; Weiser et al., 2005; Breitling et al., 2009, 2011; Diehnelt, 2013).

For the efficient analysis of large peptides libraries, an automated computer-based analysis of experimental results is crucial. It consists of a quantitative analysis of activity measurements (e.g., to compute activity-related values based on luminescence measurements of a dilution series Mikut, 2010) and the generation of new promising candidate sequences for a synthesis. The latter step can be done (i) based on a systematic substitution of single amino acids in a sequence or (ii) the computation of molecular descriptors of a sequence followed by a model-based evaluation of activity predictions (Cherkasov et al., 2009; Mikut and Hilpert, 2009; Torrent et al., 2011; Fernandes et al., 2012; Müller et al., 2016). Only few methods exist for the prediction of toxic effects to get an early insight in the therapeutic potential, see for example Cruz-Monteagudo et al. (2011). However, many relevant experimental procedures, e.g., for a detailed analysis of blood interactions (Yu et al., 2015), are not yet established for high-throughput screens. In this review, we will focus on screens for (AMPs) synthesized by the SPOT-synthesis technique.

## Peptide SPOT-synthesis

The basic concept of SPOT-synthesis is based on the capacity of reactions to run until completion in a porous and planar surface when enough reagents solution that the membrane is able to absorb is added. This observation was made separately for combinatorial nucleotides and peptide synthesis (Frank et al., 1983; Frank and Doring, 1988). Therefore, when a small droplet of liquid is dispensed on a porous membrane substrate and low volatility solvents containing reagents are used, the circular spot formed by the droplet absorption acts as an open reactor. Using this principle a great number of reactions can be arranged as arrays on a larger surface as firstly described by Frank et. al. (Frank, 1992).

Peptides produced by the SPOT-synthesis can be used in surface- and solution-phase bioassays. Parallel peptide assembly on planar surfaces using the SPOT-technique comprises some general steps that will be described in further detail: (i) selection of a membrane and its functionalization to meet the chemical, biological, and technical requirements of the synthesis and screening method; (ii) attachment of spacers and/or linkers; (iii) peptide synthesis by conventional solid-phase Fmoc-chemistry (Merrifield, 1963); (iv) cleavage of side chain protecting groups; and optional (v) peptide cleavage from the membrane for analysis and liquid-phase bioassays.

Cellulose membranes are widely used as porous material for the SPOT-synthesis. Cellulose is an inexpensive flexible, hydrophilic material, resistant to the organic solvents and basic conditions used during peptide synthesis. Moreover, it is also stable in aqueous conditions and not toxic being appropriate to be used in biochemical and biological assays. Cellulose filter papers are available in almost all laboratories and different types of these commercially available filters have been described to be suitable for the use by SPOT-synthesis, namely Whatman Chr1, Whatman 50, or Whatman 540 (Hilpert et al., 2007a; Lacroix and Li-Chan, 2014). TFA-soluble cellulose membrane can be used to obtain soluble peptide-cellulose conjugates that can be subsequently spotted onto glass slides to produce CelluSpots microarrays (Chenggang and Li, 2009). A series of alternative materials have been proposed as substrates when non-compatible polysaccharide chemistry needs to be performed: Polyvinylidene difluoride (*PVDF*), nitrocellulose, polytetrafluoroethylene (PTFE/*Teflon*), acrylate-coated PTFE, polystyrene-grafted PTFE. Many types of optimized membranes are commercially available from AIMS Scientific Products. Deiss et.al. have recently reported patterned deposition of Teflon on paper allowing parallel flow-through peptide synthesis on paper that are not possible with standard membranes where the relationship between spot size and solution volume limits the volume that can be deposited onto the support (Deiss et al., 2014).

Cellulose membranes possess hydroxyl groups at the surface with low reactivity. Arrays of spot reactors providing suitable anchor functions for peptide coupling are often obtained by esterification of the hydroxyl groups. The most commonly utilized derivatization of cellulose consist in the coupling of beta-alanine-OH due to the molecules flexibility and linear structure (Weiser et al., 2005). Higher functionalization with subsequent potential higher yield has been described with glycine functionalization (Kamradt and Volkmer-Engert, 2004). Alternatives to cellulose esterification has been also proposed as the use of 2,6-dichlorobenzoyl chloride (Sieber, 1987), the activation of the amino acids with MSNT (1-(mesitylene-2-sulfonyl)-3-nitro-1,2,4-triazole) (Blankemeyermenge et al., 1990) or the Mitsunobu-reaction (Barlos et al., 1987). Fmoc-amino acid fluorides have been shown to be highly reactive, but their synthesis is time-consuming and arginine cannot be produced (Wenschuh et al., 1999). Furthermore, they are not stable enough during SPOT-synthesis. P-hydroxymethyl-benzoic acid (HMBA) has also been proposed as a orthogonal safety-catch linkage suitable for peptides, peptoids and carbohydrate-peptide conjugates (VolkmerEngert et al., 1997).

Some protein domains require a free carboxyl-terminus (C-terminus) for ligand recognition. Peptides synthesized according to the standard SPOT-synthesis protocol lack a free C-terminus due to the coupling to the cellulose support. The approach proposed by Licha et al. to produce peptides with free C-terminus uses an Fmoc-amino acid 3-bromopropyl esters and mercapto-functionalized cellulose membranes (Licha et al., 2000). Alternatively, peptides with free C-terminus have been successfully produced by reversing the peptide orientation (inverted peptides) (Boisguerin et al., 2004). The pioneering work of Kania et al. (1994) for free C-terminal peptide synthesis on solid phase have been recently adapted to produce a 340-member library of peptides containing free C-termini on cellulose membranes (Wang and Distefano, 2012). A different strategy using 1,10-carbonyl-di-imidazole (CDI) or 1,10-carbonyl-di-(1,2,4-triazole) (CDT) activators has been also reported as an convenient approach to produce peptides with free C-terminal end by SPOT-synthesis (Ay et al., 2007a).

The size of the spots on the support is defined by the dispensed volume, the physical properties of the membrane surface and the solvent(s). The spot size together with the minimum distance between the spots limits the number of peptides that can be synthesized per membrane area (Tong et al., 2002; Tonikian et al., 2008). The SPOT-technique is particularly flexible regarding peptide number that can be synthesized. In addition, there is no special equipment needed although the laborious process of the manual synthesis (Hilpert et al., 2007b) make it only feasible when the aim is to produce a small number of peptides. Manual SPOT-synthesis kits with a membrane large enough to fit 96 spots are commercially available from Cambridge Research Biochemicals and Sigma-Genosys. Process automation allows the synthesis of up to 8000 different peptides on a 19^*^29 cm single sheet. Semi-automated and fully-automated synthesizers for SPOT-technique are commercially available, for example Intavis Bioanalytical Instruments and MultiSynTech.

Fmoc-chemistry is used for the SPOT-technique with two possibilities for the preparation of the coupling solution containing activated amino acids: *in-situ* activated or already pre-activated Fmoc-protected amino acids. For amino acids *in-situ* activation, an activator and a coupling reagent are added to the no activated Fmoc-protected amino acid derivative. The activations can be carried out by almost all known procedures: DIC/HOBT; HATU, HBTU, or TBTU with bases as DIPEA, PyBOP/DIC, or EEDQ. Pentafluorophenyl (OPfp) amino acids derivatives are normally used for the pre-activation strategy, which makes the preparation of coupling solutions very simple. However, only particular pre-activated amino acids (i.e., all common L-amino acids) are commercially available and the pre-activated derivatives of nonstandard amino acids would need to be synthesized or *in-situ* activation can be used instead. In both methods, 0.2–0.5 M solutions of Fmoc-protected derivatives are generally dissolved in NMP that are subsequently deprotected with 20% piperidine solutions. Capping steps by acetylation after each cycle are highly recommended to assure that inefficient couplings only gives rise to truncated sequences. Bromophenol blue (BPB) staining as free amino indicator allows the visual monitoring of the proper performance of the synthesis steps, such as correct dispensing, coupling, capping and effective removal of piperidine from Fmoc-deprotection steps.

When using Fmoc/tBu strategy for the peptide synthesis, side chain deprotection by concentrated TFA treatment will be required. When cellulose membranes are used, the cleavage treatment regime for resin solid-phase peptide synthesis (i.e., around 90–95%TFA for 2–4 h) would not be feasible due to the cellulose degradation at high acid concentrations. Cellulose would last for about 3 h at 50% TFA concentration or around 1 h at 90% (Hilpert et al., 2007a). To overcome that problem, several cleavage cocktails and incubation times have been proposed and reviewed previously (Hilpert et al., 2007a). Short incubations at high TFA concentrations followed by longer reactions at around 50% TFA used to give good results even for the Pbf arginine protecting group that need normally higher TFA concentration to be cleaved (Hilpert et al., 2007b).

Peptide release from the membrane can be achieved by the cleavage of the peptide C-terminal bond and the cellulose membrane. Hydrolysis at high pH can be used for peptides coupled by ester bonds. Treatment of ester linkage from HMBA, glycolic acid or similar with 10 mM sodium hydroxide give the peptide free acids. The treatment of the dry membranes with ammonia vapors yields soluble peptide carboxamides. Reported cleavage strategies has been also extensively reviewed (Wenschuh et al., 2000; Frank, 2002; Hilpert et al., 2007a).

One of the main advantages of the SPOT-synthesis is the molecule versatility that can be produced. The chemical and technical performance has been optimized for the assembly of peptide structures including: linear, cyclic, branched molecules including D and L, coded and noncoded amino acids derivatives and commercially available building blocks even for non-peptidic compounds. Moreover, the peptides can be labeled for detection purposes or for surface immobilization (Toepert et al., 2003; Winkler and McGeer, 2008).

SPOT-synthesis is based on conventional solid-phase synthesis and the same problems are observed. The quality of SPOT-synthesized peptides has been extensively investigated and peptide purity between 50 and 96% were reported. HPLC analysis of SPOT-synthesized short peptides of up to 15 amino acids showed similar purities to those synthesized by solid-phase methods in reactors (Wenschuh et al., 2000). Peptide purity higher than 92% has been reported by Takahashi et al. (2000), while when a huge number of SPOT-synthesized cytomegalovirus deduced nonameric peptides where analyzed by HPLC/MS peptide purity in the range of 50–85% was found (Ay et al., 2007b). Purities of 74.4–91.3% has been reported by Molina and co-workers (Molina et al., 1996). Even longer peptides, such as the 34-meric FBP28 WW domain could be SPOT-synthesized with a high quality of 65% purity (Przezdziak et al., 2006) or 38mer peptides described by Toepert et al. (2001). Besides the high synthetic peptide quality, equivalent peptide array quality is achieved by applying identical chemical conditions during array synthesis. Therefore, taking peptide arrays from the same cellulose membrane (intra-membrane arrays) is highly recommended. As far as possible, this would ensure spots with similar peptide density (peptide concentration in a spot).

## Screening of peptides synthesized by SPOT-technology for antimicrobial activity

### Screening of soluble peptides

To our best knowledge the first publication combining peptide synthesis of AMPs on cellulose by SPOT-synthesis and antimicrobial screen was published in 2005 by Hilpert et al. (2005). Here the authors were investigating a linear variant (RLARIVVIRVAR) of the cyclic 12mer peptide bactenecin (RLCRIVVIRVCR). Bactenecin is a peptide discovered in bovine neutrophils (Marzari et al., 1988), active against Gram-negative and some Gram-positive bacteria. The linear variant Bac2A showed similar activity profile, but somewhat improved toward Gram-positive bacteria (Wu and Hancock, 1999). The minimal inhibitory concentration (MIC) against *Pseudomonas aeruginosa* was determined in Mueller-Hinton broth at 50 μg/ml (Wiegand et al., 2008). In order to gain a high yield the cellulose membrane was modified with glycine (Kamradt and Volkmer-Engert, 2004). The synthesis was performed via Fmoc-strategy. Side chain deprotection was performed by using 90% trifluoracetic acid, 3% tri-isobutylsilane, 2% water, 1% phenol in dichlormethan for 30 min, followed by a second treatment for 120 min with 50% trifluoracetic acid, 3% tri-isobutylsilane, 2% water, 1% phenol in dichlormethan. The cleavage of the peptide from the membrane was performed by ammonia gas atmosphere using an overnight incubation.

In order to make the screen as sensitive and fast as possible the authors developed an assay where a luminescent strain of *P. aeruginosa* (H1001) was used, containing the luciferase gene cassette luxCDABE that was incorporated into the bacterial chromosome (into the fliC gene) (Lewenza et al., 2005). The authors demonstrated that the traditional time kill assay and this new developed assay are very similar in their result. In recent years, we have demonstrated that luminescence is not required to use these cellulose-derived peptides. In several projects, we have used unmodified bacteria to perform this screen with other live/death stains.

The peptide Bac2A was systematically investigated by changing each single position with 20 most occurring amino acids in nature. This substitution matrix resulted in 228 unique peptides (12 positions x 19 alternative amino acids). As a negative control a peptide with no antimicrobial activity was used (GATPEDLNQKLS-NH_2_). Each peptide was stepwise diluted seven times (1/2 the concentration each) and the activity against *P. aeruginosa* H1001 was determined. From this concentration curve, an IC_50_ was determined and in relation to the positive control (Bac2A) a proxy IC_50_ was calculated for each of the peptides. Based on this data, for each position of Bac2A, the effect of each amino acid substitution could be measured. For example, positions 1 (R), 4 (R), 8 (I), 9 (R), and 12 (R) are optimal occupied, since no other substitution, except for cysteine at some positions, improved activity, in fact most of the substitutions drastically reduced the activity. It also confirm the importance of the positive charge and that the hydrophobicity can be achieved by different amino acids. In contrast, position 11 (A) showed many substitutions that strongly improved the activity. Overall, the substitution of C, W, R, K, and H often improved antibacterial activity, whereas A, D, E, and P never improved the activity. Peptides that are synthesized by the SPOT-technology are normally not purified, and the results need to be confirmed with purified peptides. The authors have, based on the substitution matrix, designed 11 single substitution variants of Bac2A, 4 multiple substitution variants and 5 Bac2A-derived 8mers with multiple substitutions. These peptides were synthesized on resin and HPLC-purified and then the MIC against three Gram-positive, three Gram-negative (including *P. aeruginosa*) and one yeast was determined. The MIC values of the purified peptides were compared to the IC_50_ values of the crude peptide (SPOT-technology) and a good correlation of *R* = 0.895, *P* < 0.01 by ANOVA was reported. That supports the observed IC_50_ data of the control peptide Bac2A that was synthesized and tested at several different syntheses and showed very robust data for 50 replicates with 0.13 ± 0.04. There were three peptides with single substitution found that improved the MIC against *P. aeruginosa* from 50 to 8 μg/ml and two of the multiple substitution peptides reduced the MIC to 2 μg/ml. It was also confirmed that introducing a proline in the sequence decreases activity to >250 μg/ml. One 8mer peptide also showed promise with an MIC of 8 μg/ml against *P. aeruginosa*. The detailed method for producing peptides on cellulose sheets and the use of luminescent bacteria to screen for (AMPs) synthesized on cellulose was published in Nature Protocols 2007 (Hilpert and Hancock, 2007; Hilpert et al., 2007b). Jenssen et al. used this data to evaluate different descriptors for the design of (AMPs) with enhanced activity (Jenssen et al., 2007). The best outcome was a correct predicted activity that reached 84%.

In 2006, a publication described the very same synthesis and screening approach to investigate possible optimization strategies in more detail (Hilpert et al., 2006). Peptides were synthesized on cellulose with a glycine linker to gain high yield, side chain deprotection and cleavage of the peptide from the membrane was performed as described before. In our opinion, there were three important observations described: First, substitution analysis as a tool to optimize an antimicrobial peptide was confirmed. Second, even in this very flexible 12mer peptide a substitution in one position of the peptide effects distant positions. In this example, the substitution of position three influences position 11. Third, neither the primary sequence of the peptides nor the composition of amino acids alone determines the antibacterial activity for these short (AMPs). This was shown by 49 scrambled variants of the 12mer Bac2A (RLARIVVIRVAR-NH_2_) that indicates the whole bandwidth of activity from non-active to superior active. The data shows that there is one or more hidden features that also contribute to the antibacterial activity. In a quantitative sequence activity relationship study using a computational analysis and descriptors that translates sequence ordering and fragment-based hydrophobicity into meaningful numbers, a hydrophobic patch was discovered that was able to classify the peptides. In addition, circular dichroism (CD) revealed that the interaction with liposomes consisting of PPG/POPC 1:1 in 10 mM Tris buffer pH7.4 induced a strong structural change in the spectra compared to only buffer (random structure profile). These changes occurred in the active peptides but not in the less active peptides. Similar results obtained by a membrane depolarization assay using *E. coli* strengthened the data obtained by CD spectra, showing strong and fast depolarization with active peptides and only weak and slow depolarization with less active peptides. The hydrophobic patch, CD and depolarisation hint that the interaction with the membrane is a hidden feature that influences activity and is hard to predict based on the sequence. In this publication two peptides (VRLRIRVRVIRK-NH_2_ and KRWRIRVRVIRK-NH_2_) showed an MIC value of 3 μg/ml against *P. aeruginosa* and 0.8 μg/ml against *S. epidermidis* (in Mueller-Hinton-broth).

**Figure 1:**
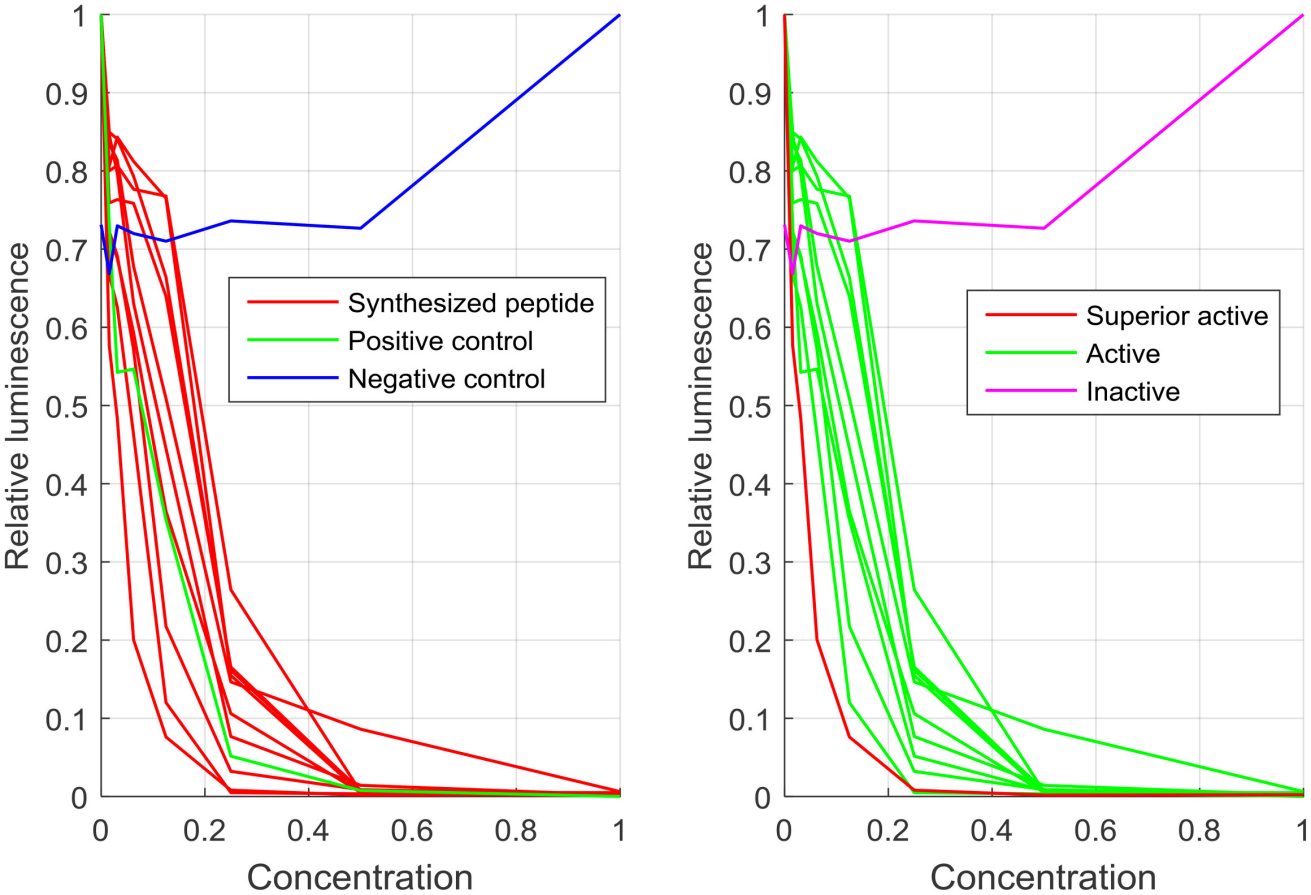
**Luciferase assay measurement of 10 peptides and two controls of library 5BM placed on plate 5 of the screen**. In the left subfigure, the luminescence values of the positive and negative control on this plated are shown compared to the synthesized peptides. The right subfigure shows the same data with the color-coded activity classification. One peptide sequence with very low luminescence values even for low concentrations was marked as superior active. The concentration and the luminescence values were normalized to the maximum values of a dilution series. The classes were defined based on RelIC_75_ values. As an example, the negative control that oscillates between relative luminescence values between 0.7 and 1 for different concentrations was classified as “inactive.”

The synthesis of hundreds of peptides via the SPOT-technology and a direct cell based screen resulting in activities ranging from totally inactive to highly active provides an optimal training set for computational analysis and consequent peptide design. Another advantage is that newly computer designed peptides can be synthesized and screened in high numbers to provide confidence in the design rules. In 2009, two publications described that approach (Cherkasov et al., 2009; Fjell et al., 2009). Three 9mer libraries were synthesized and screened against *P. aeruginosa* in the previous described screening assay using the luminescent strain H1001. The peptide libraries were synthesized on Whatman 50 cellulose membranes using glycine as a linker. Side chain deprotection procedure and membrane cleavage protocol remained the same to what was reported before. The first library consisted of 200 computer designed totally random peptides, where each amino acid (except cysteine that was excluded) had the same chance to be incorporated. The screening result showed only inactive or weak active peptides. In consequence, a second library was designed based on occurrence of amino acids in short natural occurring (AMPs). This library contained 943 members and besides inactive and weak active peptides, 26% had similar activity to Bac2A and 2.3% were superior to Bac2A. Based on this data a third library with 500 members was designed using an optimized parameter for the probability of amino acids to be selected in the computer design of new AMPs. This library was synthesized and screened as described before and the antimicrobial performance improves, 48% were similar active to Bac2A and 5% are superior to Bac2A. Thus, the parameter of the third library was used to computer generate 100,000 peptide sequences. The data of library two and three were used to train a QSAR model using “inductive” chemical descriptors and an artificial intelligence approach based on artificial neural networks. These descriptors take into account all atoms of the peptides, including hydrogen and are sensitive to the three-dimensional structure of the peptides. Therefore, all 100,000 peptides needed to be modeled by estimating structural conformation based on energy minimization in the gas phase using MMFF94 force field. The QSAR model predicted the activity of all peptides, ranked them and grouped them into four quartiles, 25,000 in each. The first 50 of each quartile were then selected, synthesized on cellulose and screened against *P. aeruginosa*. The correlation coefficient between the measured and predicted relative IC_50_ values was *r*^2^ = 0.986 (linear regression) supporting the accuracy of the model. This correlation was also confirmed by 20 selected peptides that were HPLC purified (>95%) and MIC values against different pathogens were determined. Even though, the quartiles had extremely different antibacterial activity, peptides from each quartile showed similar hydrophobicity, charge and amphipathicity/hydrophobic moment, supporting previous results about a hidden parameter that also influences the activity (Hilpert et al., 2006). Two peptides were selected and tested against a series of multidrug-resistant (MDR) “superbugs,” including MDR *P. aeruginosa*, methicillin-resistant *Staphylococcus aureus* (MRSA), extended spectrum β-lactamase producing *E. coli*, vancomycin-resistant *Enterococcus faecalis* and *faecium* (VRE) and *Enterobacter cloacea* with derepressed chromosomal β-lactamase. Most MIC values were between 0.8 and 12, especially peptide HHC-10 performed very well. Overall the peptides did outperform all tested conventional antibiotics. The highest MIC values were observed for 2 VRE isolates, with values of 99 and 49 μM, while other VRE isolates showed MIC values between 1.5 and 12 μM. The peptide HHC-10 performed also well in an intraperitoneally (IP) *S. aureus* infection in a murine model, significantly reducing the bacterial load after 24 h under both administration route, IP and IV at 4 mg kg^−1^. While the QSAR model was extremely effective, its complexity was such that an understanding of the rules for activity was impossible. The data of the three libraries was therefore re-analyzed using simpler to understand descriptors in order to finally find understandable rules that define why a short peptide is antibacterial or not. Mikut et al. answered that question in a massive amount of synthesis and screening work (Mikut et al., 2016). With an unprecedented number of individual peptides synthesized and screened the authors have showed that even elusive rules can be discovered and used to improve antimicrobial activity. For that more than 3,500 individual peptides, that is more AMPs than stored in the APD3 data base, were synthesized and tested against *P. aeruginosa*, showing the power of the SPOT-synthesis technology. Library AA0 verifies that the right amino acid composition is important, but not enough to explain the activity. Library SR uses optimized amino acid composition from AA0 and was further restricted to contain at least three positive charges and at least two tryptophan. The results, see Table 1, show that about 75% of these peptides are active, indicating the importance of a balance of charge and hydrophobicity. Library BM showed that a computer model with only one descriptor is not enough to describe activity, but five models combined (library 5BM) achieve this with about 97% accuracy, using simple descriptors only. As an example, dilution series of 10 peptides in this library are shown in Figure 1.

**Table 1:** **Overview of eight peptide libraries**.

**Library**	**Number of peptides**	**Number without outliers**	**Superior active**	**Active**	**Weak active**	**Inactive**
1	200	185	0 (0.0%)	0 (0.0%)	98 (53.0%)	87 (47.0%)
2	943	928	35 (3.8%)	163 (17.6%)	635 (68.4%)	95 (10.2%)
3	500	493	15 (3.0%)	132 (26.8%)	302 (61.3%)	44 (8.9%)
AA0	600	599	0 (0.0%)	274 (45.7%)	302 (50.4%)	23 (3.8%)
SR	600	598	9 (1.5%)	448 (74.9%)	141 (23.6%)	0 (0.0%)
BM	600	598	6 (1.0%)	360 (60.2%)	232 (38.8%)	0 (0.0%)
5BM	600	593	46 (7.8%)	533 (89.9%)	14 (2.4%)	0 (0.0%)
EP	600	597	1 (0.2%)	168 (28.1%)	283 (47.4%)	145 (24.3%)

*Number of peptides analyzed and associated activity classes against Pseudomonas aeruginosa. Outliers–peptides with implausible luminescence measurement values–were excluded from subsequent analyses. All libraries were described before [(Cherkasov et al., 2009; Fjell et al., 2009; Mikut, 2010)]*.

Library EP looks at “exotic” peptides that were poorly described by the other models. Comparing 5BM and SR revealed that all weak active peptide had either too much or too little W or R/K, respectively. In 5BM, this balance was more enforced. In order to proof that the balance is an important feature, another library with all combination of W and R in a 9mer peptide (512 peptides) was synthesized and tested, verifying that the “right” balance leads to activity. It was shown that there is no positional preference for amino acids. In addition, these short AMPs differ from those that occur naturally. The most active three AMPs identified from these libraries showed MIC values of 2.2–2.7 μmol l^−1^ against *P. aeruginosa* (in Mueller-Hinton broth). They also showed broad spectrum activity as the other 9mer peptides described before.

Two publications describe the use of SPOT-technology to optimize proline rich antimicrobial peptides (PrAMPs) (Knappe et al., 2016b). The peptide oncocin, a peptide isolated from Oncopeltus fasciatus (large milkweed bug) is a 19mer peptide with rather weak activities against *Staphylococcus aureus* and *Pseudomonas aeruginosa*, however it was successful in a systemic septicaemia infection model in mouse (Knappe et al., 2012). A substitution analysis of oncocin was synthesized on a cellulose membrane using the SPOT-technology, using glycine as a linker amino acid to improve the yield. The same side chain cleavage procedure and membrane cleavage procedure was applied as previously described. In total 361 variants of oncocin (VDKPPYLPRPRPPRRIYNR-NH2) were synthesized and screened against a luminescent *P. aeruginosa* strain (H1001). The screening was performed in 6.25% Mueller-Hinton-Broth (1.3 g/L) containing 40 mmol/L glucose, since oncocin is not active in full media. The MIC determination for selected peptides against *S. aureus* and *P. aeruginosa* was performed in 12.5% Mueller-Hinton-Broth. Analysis of the data showed that 25 substitutions at nine different amino acid positions increased the activity, whereas 86 substitutions led to a complete loss of activity. The MIC data revealed that oncocin is very robust toward substitutions of single amino acids, no strong change in activity was observed. There was however double substitutions that indeed change the activity strongly, against *P. aeruginosa* a 10 times improvement was observed resulting in an MIC value of 4–8 μg/mL and against S. aureus a 100-fold more active variant than the original oncocin was discovered showing an MIC value of 0.5 μg/mL.

**Figure 2:**
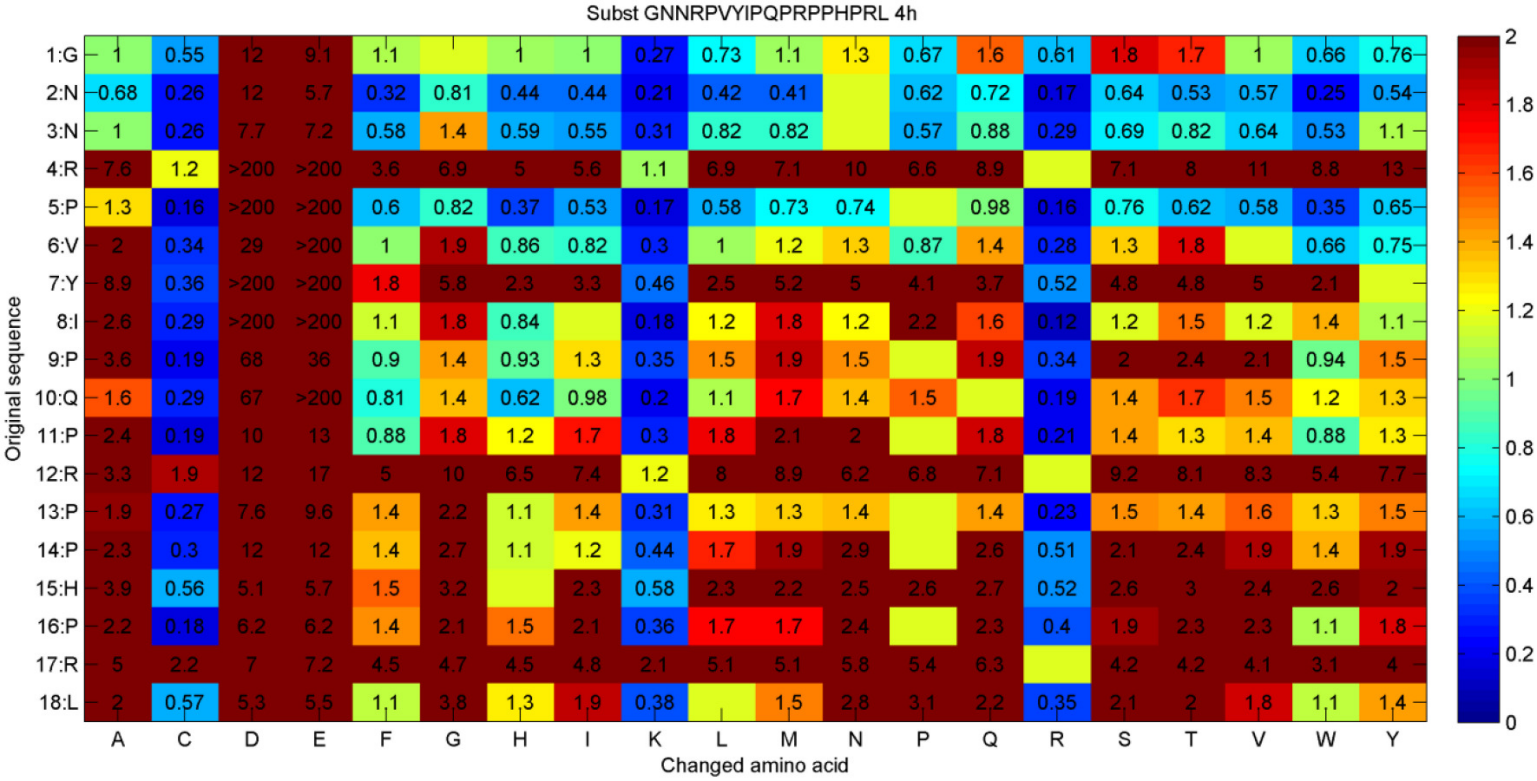
**Substitution analysis for Apidaecin (GNNRPVYIPQPRPPHPRL)**. The original sequence and amino acid positions are given in the first two columns. The other rows (A–Y) identifies the amino acid replacements at each position. Each box in the matrix corresponds to a single peptide containing an additional glycine at the C-terminus. The values within each box represent a RelIC_75_ value, determined by treatment of the *Pseudomonas aeruginosa* reporter strain H1001 with any given peptide for 4 h. Boxes are color-coded by a dynamic range between blue and red: blue stands for improved activity compared to the parent peptide, green for similar activity, and red indicates no activity. Empty boxes represent the original sequence.

A second PrAMP was investigated using the same approach. Apidaecin is an 18mer peptide that was isolated from the honey be (*Apis mellifera*). Apidaecin and its variant were also successfully used in mouse models (Czihal et al., 2012). Apidaecin (GNNRPVYIPQPRPPHPRL-OH) is inactive against *S. aureus* and very weakly active against *P. aeruginosa* (500 μg/ml in 1/8 Mueller-Hinton broth). The aim of the project was to improve these activities (Hoffmann et al., 2012). For the substitution analysis, 341 unique peptides were synthesized via the SPOT-technology and tested against *P. aeruginosa* in 1/8 Mueller-Hinton broth. The result of this analysis is presented in Figure 2.

The substitution analysis reveals that an additional positive charge improves the activity nearby at each position. Such generalized results can be identified by looking for more active variants and the manual interpretation of similarities of the related amino acids, here caused by lysine and arginine indicating the positive charge. In accordance with this, a negative charge would decrease the activity dramatically, see columns for aspartic acid and glutamic acid substitutions. In addition, the N-terminal part can still be optimized, whereas the C-terminal part is quite sensitive to most substitutions. Based on this analysis, several peptides were re-synthesized on resin and HPLC purified. MIC values against three strains were determined in 1/8 Mueller-Hinton broth (MH, 2.5 g/L), results are given in Table 2.

**Table 2:** **Antibacterial activity of Apidaecin (GNNRPVYIPQPRPPHPRL) and analogs**.

**Peptide sequence**	**MIC in 1/8 of MH [**μ**g/mL]**		
	***P. aeruginosa***	***E. coli***	***S. aureus***
GNNRPVYIPQPRPPHPRL-OH	500	5	>125
GNNRPVYIPQPRPPHPRL-NH2	250	1.25	>125
GWNRPVYIPQPRPPHPRL-NH2	64	1.25	63
GRNRPVYIPQPRPPHPRL-NH2	64–128	0.625	32
GNNRCVYIPQPRPPHPRL-NH2	125	10	31
GNNRRVYIPQPRPPHPRL-NH2	64	5	32
GNNRPVYRPQPRPPHPRL-NH2	64	0.313	63
GNNRPVYIPQPRRPHPRL-NH2	125	10	31
GNNRPVYIPQPRPCHPRL-NH2	250	20	16
GNNRPVYIPQPRPPHCRL-NH2	125	20	32
GNNRPVYIPQPRPPHPRR-NH2	125	1.25–2.5	125
GWNRPVYIPRPRPPHPRL-NH2	16–32	0.63	16
GWNRPVYIPQPRRPHPRL-NH2	64	5	4–8
GNNRPVYIPRPRRPHPRL-NH2	64	2.5	4
GWNRPVYIPRPRRPHPRL-NH2	32	2.5	2
gu-OWNRPVYIPRPRRPHPRL-NH2	4	8	8–16

*Minimal inhibitory concentrations (MIC) were determined 12.5 % Mueller-Hinton broth*.

*gu-O = (N,N,N0,N'-tetramethylguanidino-ornithine)*.

*Changes that were introduced into the parent sequence are marked in color*.

By substituting a glycine on position one to a N,N,N0,N′-tetramethylguanidino-ornithine and substituting three amino acids that were identified by substitution analysis, the activity against *P. aeruginosa* could be increased by 125-fold. By the very same substitutions (without position 1), a peptide with no activity against *S. aureus* was now highly active showing an MIC value of 2 μg/ml. This example again demonstrates the power of the method.

Bluhm et al. reported the use of SPOT-synthesis to optimize an apidaecin variant Api137 (gu-ONNRPVYIPRPRPPHPRL-OH) (Bluhm et al., 2015). Api 137 showed only activity in diluted media and variants were required that were also active in ½ MH broth. The authors were concerned about a free C-terminus and therefore changed the linker strategy to the HMBA-linker, that was reported before (VolkmerEngert et al., 1997). The first coupling at the membrane was performed with a beta-alanine. HBTU in the presence of DIPEA as base (0.2 mol/L each, 10 mL DMF, RT, 1 h) was used to couple the HMBA-linker. Fmoc-Leu-OH (0.4 mol/L) was coupled using DIC (0.2 mol/L) and DMAP (8 mmol/L) in DMF (10 mL) overnight. Benzoic anhydride (0.2 mol/L) dissolved in a mixture of pyridine (40 mmol/L) and DMF (10 mL, 2 h) were applied to cap the remaining free anchors. HBTU and NMM (0.4 mol/L each) in DMF (10 mL, RT, 2 h) was used to obtain N,N,N′,N′-tetramethyl-guanidino-groups at the N-termini. Cleavage with aqueous ammonia resulted in a mixture of free-C-terminus and amidated C-terminus as well as peptide with beta-alanine. Changing to aqueous trimethylamine changed the cleavage product to free C-terminus, however a large part of the peptides still showed the beta alanine linker as undesired side product. Api 137 purity was determined by HPLC to be 57%. The impurities were not affecting the antimicrobial activity and the screen using the complete substitution analysis was performed. The authors identified four peptides, all single substitutions that were eight times more active in 50% MH broth compared to Api137. All multiple substitutions did not result in further improvements.

The aforementioned 12mer peptides that were optimized against antibacterial activity were also investigated for their immunological properties. Based on this work K. Hilpert designed peptide libraries HH1 to HH18 and to further improve the library he designed IDR-1001 to IDR-1048. Some of these peptides were very successful in several aspects, being potent innate defense regulators and also demonstrating potent anti-biofilm activity (Wieczorek et al., 2010; Rivas-Santiago et al., 2013; de la Fuente-Núñez et al., 2014). Haney et al. explored this further with SPOT-synthesis using a restricted set of amino acids and determining anti-biofilm properties but also immune-modulatory activities (Haney et al., 2015). Two peptides were investigated, IDR-1002 (VQRWLIVWRIRK-NH_2_) and IDR-HH2 (VQLRIRVAVIRA-NH_2_) and based on this results new peptides were designed.

### Screening of tethered peptides

In 2009, a landmark publication showed that SPOT-synthesis can be used to screen and optimize surface-tethered (AMPs) (Hilpert et al., 2009). LaPorte et al. (1977) and Haynie et al. (1995) showed previously that (AMPs) can be active whilst tethered to a surface, however it was not followed up by the scientific community. The 2009 publication inspired directly and indirectly a lot of research on surface protection using (AMPs), now a field that has been reviewed on its own right. Crucial for the use of SPOT-synthesis and a screen for tethered peptides was the stability of the peptides on the membrane. An HPLC analysis of the supernatant of peptide spots, produced via the standard procedure resulting in an ester between glycine and the membrane, showed an almost completely release after 4 h incubation at 37°C in 100 mM Tris-buffer. The linker strategy was therefore changed to a N-CAPE linker, a strategy that allows with further modification the synthesis of peptides with free C-terminus (Licha et al., 2000; Bhargava et al., 2002). This N -modified cellulose-amino-hydroxypropyl ether provided very stable tethered peptides, showing no HPLC detectable traces after 4 h incubation at 37°C in 100 mM Tris-buffer. Peptides were synthesized at 50 nmol/spot and 200 nmol/spot. In total, 122 tethered peptides were screened and 23 highly active peptides were identified. These peptides were selected on their ability to kill bacteria in solution. There was no correlation observed between antimicrobial activities of tethered peptides compared to the MIC of the peptides in solution. There was however the observation that the 10 most active peptides on an MIC level were also highly active when tethered. It was also shown that the haemolytic activity of the peptides dropped once tethered to a surface. The activity of the antimicrobial activity of selected peptides were confirmed using other surface linking chemistry and other types of surfaces. Several experiments were performed to unravel the mode of action of these peptides. In a follow up study, several of these peptides were attached to a titanium surface using a copolymer brush (Gao et al., 2011). After the characterization of the surface the antimicrobial activity was tested and verified in a rat infection model.

## Summary and outlook

Antimicrobial resistance is a natural phenomenon that is part of microbial surviving strategies to secure resources and ecological niches. Alexander Fleming already said: “It is not difficult to make microbes resistant to penicillin in the laboratory by exposing them to concentrations not sufficient to kill them, and the same thing has occasionally happened in the body. The time may come when penicillin can be bought by anyone in the shops. Then there is the danger that the ignorant man may easily underdose himself and by exposing his microbes to non-lethal quantities of the drug make them resistant.” Unfortunately, man became extremely ignorant and careless and started to misuse all the antibiotics on a large scale, accelerating the drive of resistant organism. Antibiotics are misused in a metric ton scale in animal farming, for example treating piglets to get fatter faster and less ill under the terrible condition they are kept in mass farming. A public awareness campaign is trying to change this mind-set, however the next generation faces a very difficult time with not much treatment options left for bacterial and fungal infections. Often it is referred to as the return to medieval medicine. This situation becomes more likely since the economic prospects to develop novel antibiotics for pharma are rather bleak and many companies dropped out from their antimicrobial drug development program, 36 of them last year. That leads to very little activity in this sector, for example last year 504 drug candidates entered clinical phase 2 and 3 studies for cancer treatment, but only 37 for antimicrobials.

Antimicrobial peptides (AMPs) are possible new candidates for the treatment of MDR bacterial infections since they are able to kill MDR microbes. It is a very diverse class and already different modes of actions were reported for different peptides, making them very interesting as drugs with novel mode of action. There is a substantial body of literature about antimicrobial action and their immunomodulatory activities. There is however a great lack of data about the translational aspect toward clinical phase and this is also reflected in the few peptides that entered clinical phase. In order to make detailed studies of these peptides, a method is needed that allows the synthesis of sufficient material to perform cell-based tests in a high throughput manner. SPOT synthesis is used for such studies, because it is fully automated, reasonable priced and produces enough material to perform a few cell-based studies. This technique is now more than 25 years old and many data is described that shows the impact, but also a lot of chemistry to adapt the protocols to different biological questions. Recent improvements in high density peptide arrays have outperformed the SPOT technology in the field of binding assays. However, for cell based assays, were more material is needed, SPOT technology remains the lead.

Antimicrobial peptides (AMPs) were investigated using the SPOT technology. It was shown that it is possible to systematically improve the antimicrobial activity by using substitution analysis. More than 100-fold improvements in activity were reported. In addition, peptide libraries can be designed and optimized to contain very potent antimicrobial compounds. These data can be used as a base for bioinformatics and powerful prediction algorithms were developed. In the future, this technology can support the process of moving these peptides toward clinical studies, for example peptide variants and modifications can be screened for stability and activity in serum/blood.

Unfortunately, (AMPs) are currently developed not only for treatment of MDR infections in humans but also for animals and plants. Mankind seems not to learn the lessons from their ignorance but intensify their behavior. At that large scale application resistant strains can develop. Since AMPs are a major compound of the innate immune system of many organisms, including plants and animals, bacterial and/or fungal strains that will develop resistance to AMPs might threaten the ecology of earth even further and can accelerate the dying of numerous species.

## Author contributions

PL, EG, LB, RM, and KH were writing different sections to the paper. KH brought all parts together and wrote the paper in a uniform style. All authors have than proofread the manuscript and KH has finalized the manuscript.

### Conflict of interest statement

The authors declare that the research was conducted in the absence of any commercial or financial relationships that could be construed as a potential conflict of interest.
